# The role of biogeochemical hotspots, landscape heterogeneity, and hydrological connectivity for minimizing forestry effects on water quality

**DOI:** 10.1007/s13280-015-0751-8

**Published:** 2016-01-07

**Authors:** Hjalmar Laudon, Lenka Kuglerová, Ryan A. Sponseller, Martyn Futter, Annika Nordin, Kevin Bishop, Tomas Lundmark, Gustaf Egnell, Anneli M. Ågren

**Affiliations:** Department of Forest Ecology and Management, SLU, Skogsmarksgränd, 901 83 Umeå, Sweden; Department of Forest and Conservation Sciences, University of British Columbia, Vancouver, V6T 1Z4 Canada; Department of Ecology and Environmental Science, Umeå University, 901 87 Umeå, Sweden; Department of Aquatic Sciences and Assessment, Swedish University of Agricultural Sciences, 750 07 Uppsala, Sweden; Department of Forest Genetics and Plant Physiology, Umeå Plant Science Centre, Swedish University of Agricultural Sciences, Umeå, Sweden; Department of Earth Science, Uppsala University, 752 36 Uppsala, Sweden

**Keywords:** Biogeochemical hotspots, Hydrological connectivity, Landscape heterogeneity, Minimizing forestry effects, Water quality

## Abstract

Protecting water quality in forested regions is increasingly important as pressures from land-use, long-range transport of air pollutants, and climate change intensify. Maintaining forest industry without jeopardizing sustainability of surface water quality therefore requires new tools and approaches. Here, we show how forest management can be optimized by incorporating landscape sensitivity and hydrological connectivity into a framework that promotes the protection of water quality. We discuss how this approach can be operationalized into a hydromapping tool to support forestry operations that minimize water quality impacts. We specifically focus on how hydromapping can be used to support three fundamental aspects of land management planning including how to (i) locate areas where different forestry practices can be conducted with minimal water quality impact; (ii) guide the off-road driving of forestry machines to minimize soil damage; and (iii) optimize the design of riparian buffer zones. While this work has a boreal perspective, these concepts and approaches have broad-scale applicability.

## Introduction

Water quality of streams, rivers, and lakes is primarily regulated by hydrological and biogeochemical processes occurring in the contributing catchment soils. In general, although they cover a small fraction of the total catchment, areas closer to surface waters, such as riparian zones and floodplains, have a larger influence on surface waters when compared to upland soils further away (Hedin et al. 1998, Laudon et al. 2011a; Ledesma et al. 2013; Pinay et al. 2015). However, it is not the physical distance *per se* that determines the role of different landscape units for water quality. Instead, it is the hydrological connectivity between surface water and the biogeochemical sources of different solutes draining catchment soils that matters the most (Bracken et al. 2013). The dynamic pathways of groundwater draining catchments determine what areas become hydrologically connected during different runoff conditions (Jencso and McGlynn 2011) and therefore regulate biogeochemical patterns and dynamics related to both natural variability, and those caused by human perturbation (Laudon et al. 2011b).

Forestry is the dominant land-use in many forested regions of the world, and constitutes an important economic base in numerous communities, regions, and countries. More intensive biomass production is anticipated because of increasing global demand for sustainably produced lumber, paper, and energy (Berndes et al. 2003; Kraxner et al. 2013). To meet this increasing pressure of biomass production, while at the same time minimizing the negative land-use impacts on water quality, it is necessary to recognize how ecosystem services link to heterogeneity of the forest landscape. Terrestrial environments cannot be viewed as uniform entities, but instead must be regarded as mosaics of landscape elements that play distinct roles for controlling water quality. More specifically, this means forest management strategies must include mechanistic insights related to patch-specific characteristics including hydrological connectivity to surface waters, the storage and transformation of elements, and susceptibility to perturbation.

Since the 1950s, forestry has become increasingly mechanized and several types of forest machines traffic soils off-road during a rotation period. In the boreal zone, a clear-cutting system with cut-to-length logging has become the norm in many countries (Gerasimov et al. 2013; Hiesl and Benjamin 2013), where harvesters cut and limb the trees at the stump, and forwarders bring the round-wood out to roads for transport to industry. A fully loaded forwarder can weigh as much as 40 metric tons and is often the heaviest machine trafficking soils during a rotation period. Thus, following a clear-cut, besides the obvious effects of forest canopy removal (i.e., hydrological, biogeochemical and ecological changes), catchment soils are also often subject to additional off-road driving associated with wood fuel extraction, site preparation, fertilization, and thinning.

The extent to which these forestry operations cause local soil perturbation only, or also lead to downstream water quality impairment, depends on the hydrological connectivity between disturbed locations and adjacent streams. For example, some areas with sensitive soils are also hotspots of biogeochemical transformations and have high hydrological connectivity to surface water (Ledesma et al. 2013; Schelker et al. 2013). Consequently, forestry operations in such areas can result in stream water perturbations that can have detrimental long-term ecosystem effects, including soil erosion resulting in sediment transport (Kreutzweiser and Capell 2001), methyl mercury production and export that leads to downstream bioaccumulation (Bishop et al. 2009), and nutrient leakage that may cause surface water eutrophication (Futter et al. 2010).

Because of the glacial history of much of the boreal region, the hydrological conductivity of till soil, which is the predominant soil type in the northern landscapes, generally increases exponentially toward the soil surface. This well-established vertical pattern allows hydrological flowpaths to be predicted based on digital elevation models (DEM), under the assumption that topography and gravity control water movement, and that the surface of the water table follows the soil surface (Rodhe and Seibert 1999). When sufficient water converges into lower lying landscape locations, a stream is formed. The size of the converging area needed to create a stream varies spatially and temporally. The flow initiation threshold, also called accumulated area (Tarboton et al. 1991), is larger during dry conditions and smaller during snow melt and prolonged or heavy rain events which trigger the formation of intermittent or episodically activated streams. These headwater streams represent the capillaries of the forest landscape, host biologically rich communities, and serve as the primary interface between terrestrial and aquatic ecosystems (Meyer et al. 2007). In fact, a dominating proportion of all fresh- and coastal waters originate from small headwater streams, which makes them important sources of most natural and anthropogenic elements in downstream habitats (Bishop et al. 2008). At the same time, due to their large total length, headwaters often receive less protection during forestry operations (Kuglerová et al. 2014a). Inclusion of these small streams is hence of fundamental importance for the next generation of spatially explicit management plans which aim at minimizing water quality impairment.

The purpose of this paper is to summarize and synthesize our basic understanding of topographic influences on water accumulation, hydrological connectivity, and landscape sensitivity to forest management operations. Further, we discuss how this information can be formulated into what we call a ‘hydromapping tool’ that can be used to design land-use management in order to minimize water quality impact from forestry operations. We describe the benefits of using such an approach as a means of balancing tradeoffs between forest biomass yield and water quality and discuss how this can be implemented into practical operations. We focus on how the hydromapping tool can be used for three aspects of land management planning: (i) locating areas where more intensive forestry practice can be conducted with minimal impact on water quality; (ii) minimizing driving damage to wet organic soils; and iii) optimizing the design of riparian buffer zones.

## Hydromapping: A concept to minimize water quality impact

**Fig. 1:**
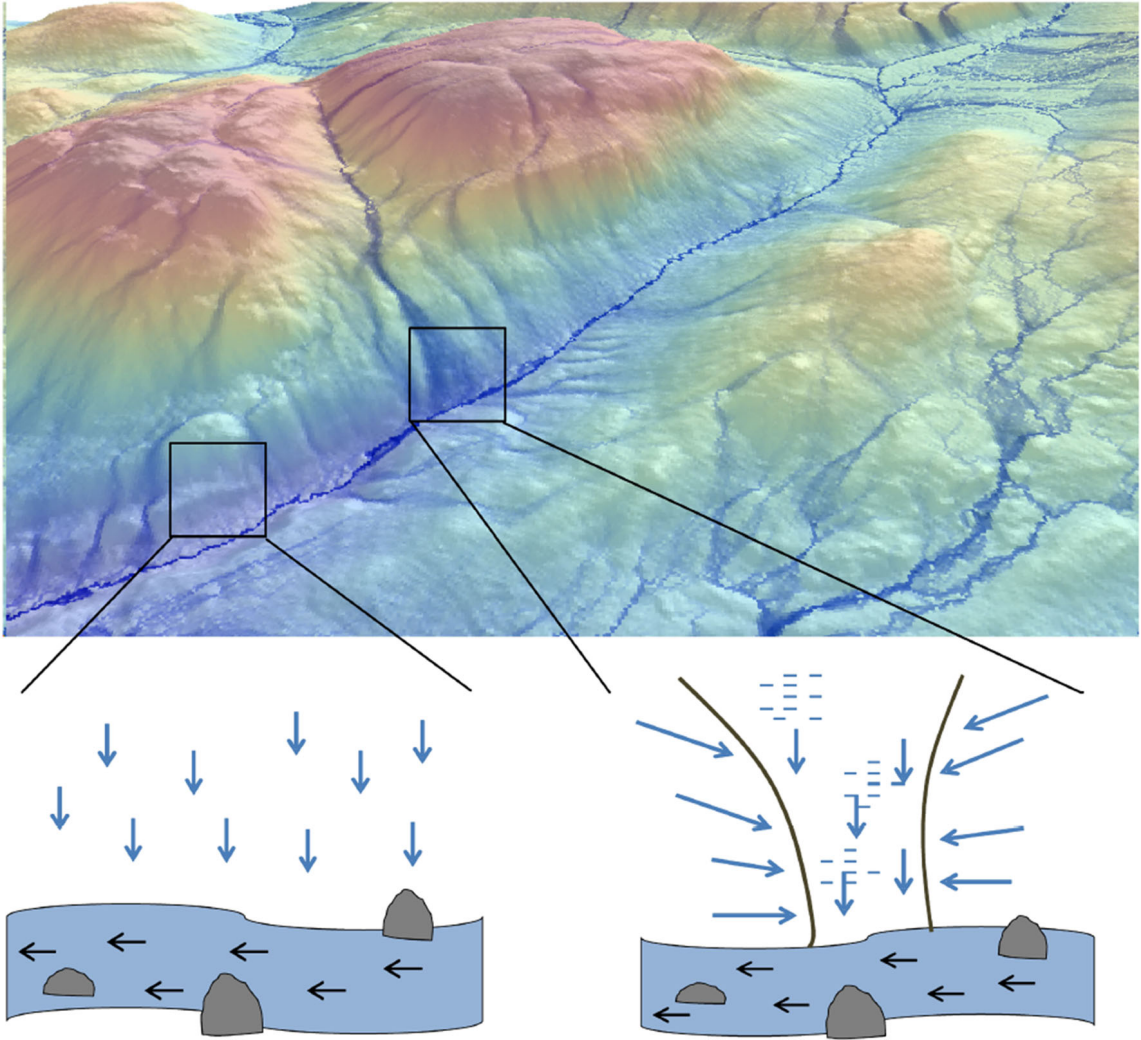
Hydromapping (*upper panel*) is derived from high-resolution digital elevation models calculating how much land area and hence water (in *blue*) that accumulates to any specific location in the landscape. This results in wet locations because of large land areas accumulating water (*lower right*) and dry locations when small amounts of land and hence water is accumulated (*lower left*). Such accumulation of water can occur in both upslope and riparian areas

**Fig. 2:**
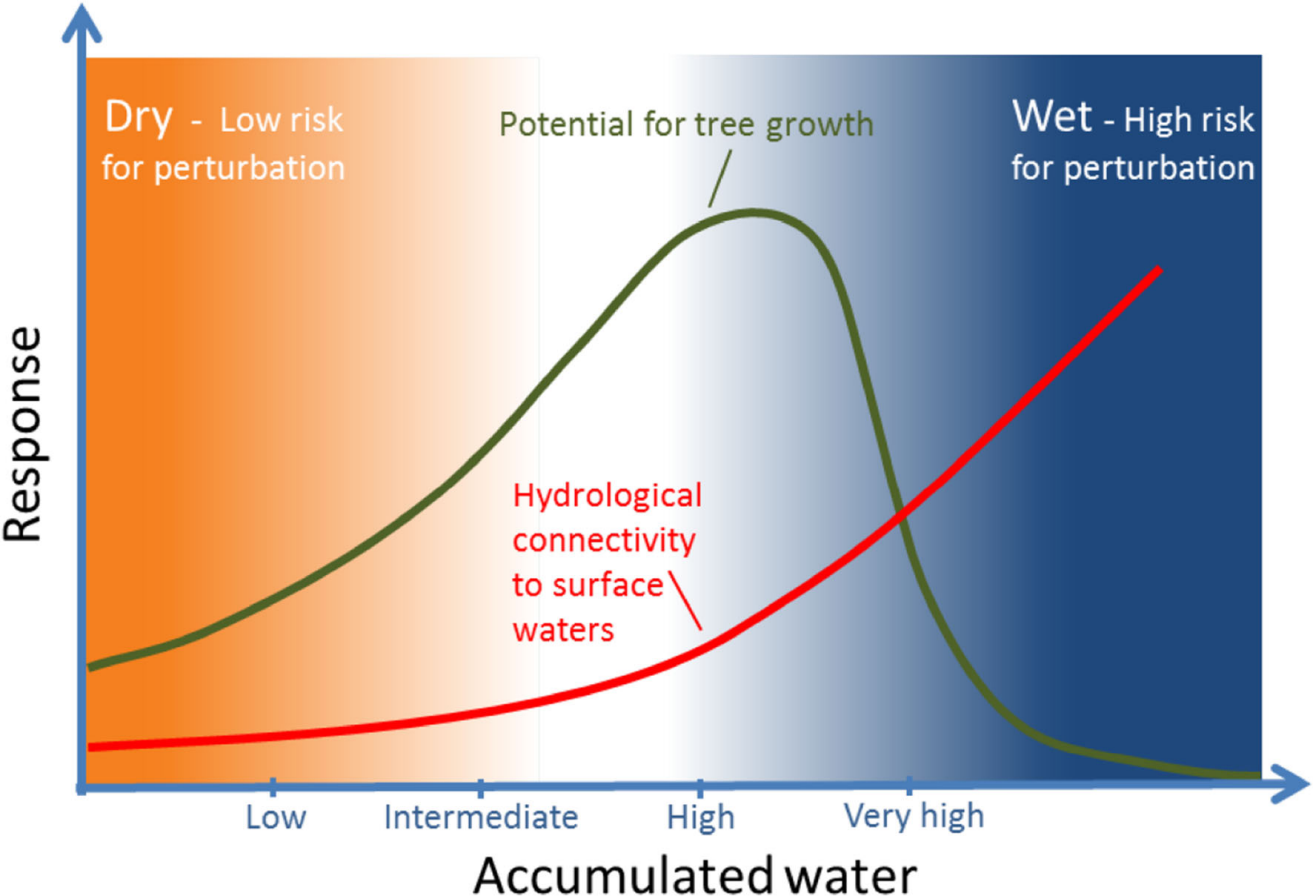
A schematic model of how the amount of accumulated water, which is calculated from the uphill area that drains into a specific location as a result of topography, effects the potential for tree growth, and the hydrological connectivity. The more accumulated water the higher is the connectivity to the stream and therefore the risk for surface water perturbation caused by different forest management activities

The landscape sensitivity framework that we call hydromapping (Fig. 1) is based on the hydrological principle that groundwater flow pathways are controlled by local surface topography (Rodhe and Seibert 1999). The convergence of local topography is hence the primary mechanism causing gradients in soil wetness, nutrient flux, and biogeochemical cycling in the landscape (Giesler et al. 1998; Zinko et al. 2005). Because topography has such a strong influence on groundwater pathways, neighboring areas can vary greatly in water storage, ranging from local conditions where tree growth is limited by lack of soil moisture, to those areas where growth is limited by too much water (Grabs et al. 2009; Murphy et al. 2011). As hydrological pathways not only control the transport of water but also nutrient and mineral solutes (Giesler et al. 1998), the spatial distribution of growth-limiting factors may follow the same general trend. Therefore, we expect the highest potential for biomass production to be found in locations that have the largest contributing areas and thus receive the most groundwater and nutrients, but still are not so flat that they become waterlogged (Fig. 2).

The strong topographic control over hydrological pathways and biogeochemical properties of soils provides the conceptual basis for landscape management. With new computational techniques and high-resolution data, the spatial distribution of groundwater flow, and thus the biogeochemically active zones, can be obtained solely from catchment topography. Indeed, the emergence of high-quality DEMs has led to the development of multiple algorithms that predict hydrological pathways and water accumulation in landscapes (Seibert and McGlynn 2007), including the topographic wetness index (Beven and Kirkby 1979), topographic position index (Weiss 2001), and cartographic depth-to-water (Murphy et al. 2008). These models allow for estimates and mapping of soil water conditions with high accuracy, and have been found to closely correspond to field observations of wetness condition (Kuglerová et al. 2014b; Ågren et al. 2014). Several studies have documented the utility of digitally derived wetness indices in predicting a variety of abiotic and biotic responses to soil moisture gradients (Zinko et al. 2006; Murphy et al. 2011). Moreover, several authors have recently argued that landscape management should rely on such tools for reducing water quality perturbation, delineating protective areas and designing riparian buffer zones (Gorsevski et al. 2008; Arp 2009; Kuglerová et al. 2014a).

Nevertheless, modeled soil wetness conditions and subsurface water flow paths have limitations. The utility of DEMs is dependent on their precision and accuracy, which often vary across and within regions. In many countries, new high-resolution LiDAR (Light Detection and Ranging)-based maps are becoming available, providing DEMs with up to 1 m cell size. Although such small-scale accuracy of the DEM can substantially improve the hydrological models and consequently hydromapping, it also introduces new difficulties related to data management, computing requirements, and selection of the right scale for calculations (Ågren et al. 2014). Furthermore, the assumption that hydrological flow paths are controlled simply by topography does not hold true for all soils. For example, on sorted sandy/gravelly soils (e.g., eskers), the soil permeability, i.e., the saturated hydraulic conductivity, instead of topography controls water flows (Ågren et al. 2014). Given these challenges, before digitally derived wetness indices can be applied to every-day planning of forest landscape management, questions of accuracy vs. data manageability and soil types need to be fully evaluated. Below we discuss how these models, together with new data, can be used operationally to locate sensitive/insensitive areas in order to optimize forest operations and forest production as well as protecting surface waters by identifying and targeting areas for different management options.

## Landscape heterogeneity and sensitivity

Since the last glaciation, water movement has been the primary regulator of soil development, vegetation patterns, and nutrient availability in the boreal landscape (Jansson et al. 2007; Ledesma et al. 2015). As topography determines water flow pathways, areas close to drainage divides and/or on convex slopes receive low amounts of accumulating water from the surrounding landscape and therefore remain dry and nutrient poor. In such areas, the lack of water limits both mineralization and weathering rates and hence nutrient availability and tree growth potential (Fig. 2). At the other extreme, the wettest landscape positions are located in topographic hollows in valley bottoms which receive water from surrounding hillslopes all year. Such areas often experience saturated conditions all year round, in which regions with a positive water balance commonly give rise to organic soil forming processes—paludification—that in northern latitudes result in mire formation. Waterlogged soils give rise to reducing conditions, which result in low mineralization rates of soil organic matter. Hence, tree growth in these constantly saturated soils is limited by a lack of oxygen, but also a shortage of plant available nutrients.

Locations in downhill concave hillslopes often experience intermediate to high accumulation of water (Fig. 2). The hydrology of such locations has created favorable conditions in the soil with more available nutrients, higher concentration of base cations, more diverse biotic communities, and greater tree growth potential (Giesler et al. 1998; Zinko et al. 2005). Provided that mineralogy and soil structure is relatively homogeneous, the main mechanism regulating the amount of mineral weathering products in soil water is groundwater residence time (Klaminder et al. 2011). This means that longer water flow paths will result in increasing concentrations of base cations and higher pH (Peralta-Tapia et al. 2015), which in turn will result in more rapid mineralization of organic nitrogen into plant available forms (Giesler et al. 1998).

Topographic position in the landscape not only shapes conditions for tree growth in a specific location, but it also determines the hydrological connection between that specific position along the hillslope and downstream surface waters. This in turn amplifies the potential for perturbation of stream water quality, as the amount of accumulated water and hydrological connectivity to surface waters both increase. While an area in close vicinity to a stream or lake can have limited impact on surface water quality because of lack of connectivity, areas further away can be more highly connected and hence be of greater importance. In the boreal landscape, the most highly connected soil-surface water areas are often organic soils in riparian zones and larger mire complexes (Grabs et al. 2012).

**Fig. 3:**
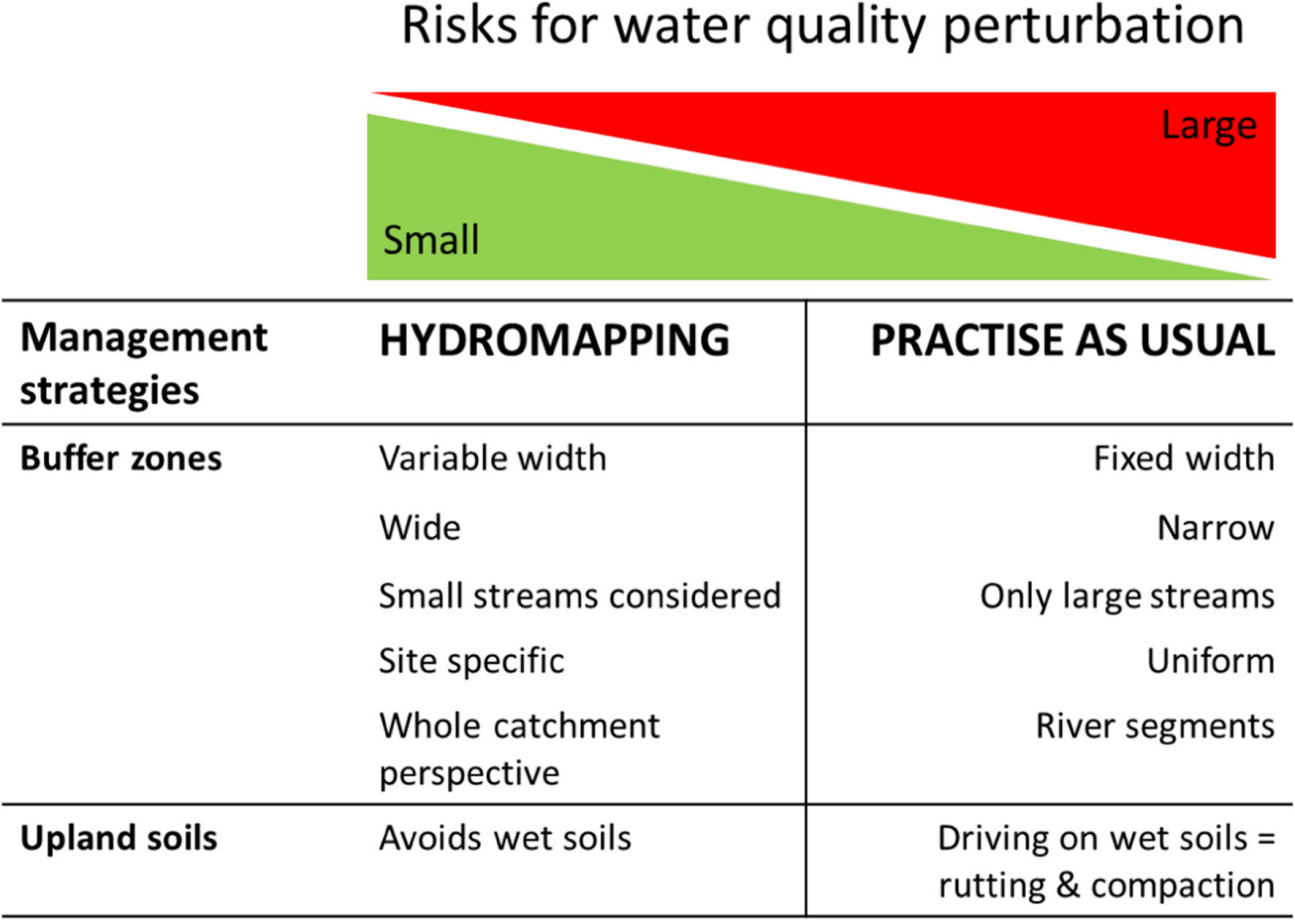
A conceptual model describing the risks for water quality perturbation associated with forest management strategies. The management strategies pose a smaller risk to water quality when hydromapping tools in riparian buffers as well as on upland catchments are considered. The risks are reduced when variable buffer widths with wider buffers at groundwater hotspots are applied, when buffers are retained along small streams and when planning is prepared using a whole catchment scale perspective including considerations of site-specific conditions. For upland soils, the risks are generally lower, but increases on wet soils with high connectivity to surface water

Wet organic soils are commonly highly connected to surface waters, and they are also biogeochemical hotspots for many elements and sensitive to physical disturbance. For example, areas at the interface between mineral and organic soils provide optimal conditions for accumulation of metals (Lidman et al. 2014) and for methyl mercury production (Mitchell et al. 2008), whereas topographic hollows are hotspots for carbon accumulation, nitrogen cycling, and plant biodiversity (Kuglerová et al. 2014a). Both soil interfaces and topographic hollows are sensitive to land-use perturbation as organic soils with high groundwater levels have low bearing capacity and are thus prone to rutting, soil compaction and other forms of soil damage caused by off-road driving (Fig. 3).

Since groundwater flow paths regulate resource availability and forest growth potential in boreal landscapes, it is possible to find areas for forest management intensification where the impact on water quality is minimized through the use of hydromapping tools. For example, areas with intermediate accumulation of water are characterized by a relatively high growth potential, but still have low connectivity to surface waters, reducing the risk for downstream perturbation of water quality. Furthermore, these areas also tend to have low connectivity to surface waters and lower plant biodiversity (Kuglerová et al. 2014b). On the other hand, areas with very high accumulation of water have lower growth potential but the risks of soil perturbation and downstream impacts on water quality associated with forestry operations are high. Taken together, we suggest that considering water accumulation and hydrological connectivity of topographically defined landscape units can result in optimizing the framework for evaluating and applying different management options to minimize negative impacts on ecosystem services.

## Minimizing driving damage

Driving on forest soils with heavy machines can affect both terrestrial and aquatic ecosystems. On land, rutting and soil compaction from forest machinery can affect root development (Schaffer and Wilpert 2012) and soil microbial communities (Frey et al. 2009) thereby altering forest growth (Miwa et al. 2004). Aquatic ecosystems can be affected when exposed mineral soils in wheel tracks are flushed into the streams causing siltation and deterioration of stream habitat, thereby decreasing the reproductive success of fresh-water fish and the diversity of macroinvertebrates (Lisle 1989). Ruts can also increase the leakage of mercury from forest soils to surface waters (Munthe and Hultberg 2004). The susceptibility of soils to compaction and rut formation depends on many different properties, for example, whether the soils are cohesive or non-cohesive, organic or mineral, and dry or wet (Saarilahti 2002). In unsorted till soils, the bearing capacity can change dramatically from one location to another. Despite this high degree of variability, systematic patterns can be seen where the bearing capacity decreases with soil moisture and organic matter content, and thus topographic position. In general, wet organic soils have a much lower bearing capacity, compared to most mineral soils (Uusitalo and Ala-Ilomäki 2013).

Protecting soils from rut formation can be done in different ways. In regions with seasonally frozen soils, driving during winter is the most common approach. However, climate change scenarios predict higher precipitation and shorter periods of soil frost, which will increase the risk of soil damage in the future for many northern regions (Jungqvist et al. 2014). Technical solutions to strengthening the soils by creating slash mats (Gerasimov and Katarov 2010) or using protective soil devices in sensitive areas will likely become more important, but also more expensive management options in the future. Another way of decreasing rut formation during forestry operations is to plan driving in such a way that the heaviest machines (the forwarders) are steered away from the wettest and most organic soils and also to avoid many passes on these the most sensitive soils (Naghdi and Solgi 2014). This is where hydromapping can provide a practical management tool by creating predictive models for soil susceptibility to rut formation that can guide harvest planning.

## Optimizing riparian buffer zones

By virtue of their physical location at the edge of streams and rivers, riparian zones often play a fundamental role in the regulation of stream water quality (Hill 1996; Grabs et al. 2012). Riparian forests in the boreal landscape also harbor substantially higher number of plant species in comparison to the upland forest floor (Nilsson et al. 2012) and are hence important for biodiversity and dispersal of organisms (Gundersen et al. 2010). Although the importance of riparian zones for water quality, quantity, and biodiversity has been acknowledged (Kreutzweiser and Capell 2001; McDonnell 2003; Sabo et al. 2005), guidelines for appropriate management of riparian forests are in need of improvement.

When catchments are harvested, it is a common practice to retain intact or marginally managed strips of trees along streams, rivers, and lakes with the assumption that tree-covered riparian buffers will mitigate negative impacts of upland forest harvesting (Castelle et al. 1994; Kreutzweiser et al. 2012; Richardson et al. 2012). Riparian buffers can help prevent sediment and nutrient loading (Kreutzweiser and Capell 2001; Feller 2005), maintain habitat for riparian organisms (Hylander et al. 2004; Biswas and Mallik 2011) and/or buffer changes in stream water temperature and light (Kreutzweiser et al. 2009). On the other hand, it has also been shown that current techniques for riparian buffer retention can be ineffective in preventing negative impacts of forestry (Spackman and Hughes 1995; Hylander et al. 2002; Broadmeadow and Nisbet 2004; Kreutzweiser et al. 2008; Lecerf and Richardson 2010). The lack of effectiveness is likely because riparian buffers are typically retained as uncut tree-covered strips with uniform width and age structure, regardless of site-specific conditions (Buttle 2002; Lee et al. 2004; Richardson et al. 2012). Uniform-width buffers have been implemented with the best intentions, but new conceptual understanding of riparian functioning, together with computational techniques and digital maps, provides forest managers with the insights and tools needed to optimize buffer designs that move well beyond fixed-width approaches (Kuglerová et al. 2014a).

It is well established that riparian soils are closely connected to surface water due to their close proximity and low topographic position in the landscape at the receiving end of groundwater movement (Fisher et al. 2004). Nevertheless, only recently, it has become clear that groundwater fluxes and water tables within riparian zones are heterogeneous across small spatial scales (Creed et al. 2011; Grabs et al. 2012), and thus, the hydrological connectivity and subsequent control of riparian soils over stream water quality vary along stream segments. If forestry aims to sustain the best possible functioning of riparian buffers, these hydrological principles need to be implemented into management (Creed et al. 2008). Indeed, the use of variable width buffer zones with different management intervention has been shown to be a promising solution for addressing tradeoffs between forest growth, biodiversity conservation, and water quality impairment (Murphy et al. 2008; Arp 2009; Kreutzweiser et al. 2012).

Riparian forest sites with large groundwater contributing areas (i.e., with wet and organic soils) are wider than adjacent drier riparian sites, and often exceed standardized buffer widths (Kuglerová et al. 2014a). As a result, the distal end of these riparian areas is often harvested and driven over when fixed-width buffers are applied. The use of wider riparian buffers for wet and hydrologically active riparian areas should thus be a better management practice. At the same time, riparian soils with lower hydrological connectivity and drier soils can represent marginal areas where harvesting closer to the stream edge does not cause adverse effects on aquatic ecosystems (Buttle 2002; Mallik et al. 2013). Further, partial and selective cutting within both wet and dry riparian buffers could be used to maintain diversity of tree age structure and canopy gaps, important aspects of forest ecology (Esseen et al. 1997), and land–water interactions (Brooks et al. 2012). In fact, such buffer designs could closely resemble natural riparian dynamics, mimicking effects of forest fires, storms, beaver activity, or insect outbreak—disturbances which would occasionally partly or completely remove riparian trees and retain riparian forests of variable widths and age structure (Buttle 2002; Kreutzweiser et al. 2012; Sibley et al. 2012). However, to minimize soil disturbance, such partial cutting would need to be performed with techniques that do not cause soil disturbance. Former silvicultural practices may have resulted in a stand structure highly susceptible to wind throws, i.e., single age class, tall conifer-dominated stands with superficial root systems. In such cases, partial cutting in buffer zones should be avoided in conjunction with clear-cutting. Emphasis on creating a wind resistant buffer zone together with other desired qualities should instead be put into regeneration, pre-commercial thinning, and thinning operations so as to favor structural heterogeneity in terms of species mixtures and tree height (Dobbertin 2002). Importantly, this spatially explicit approach to buffer management should not negatively affect economic yields, as forest production is reduced and tree composition skewed toward less valuable species (e.g., alder, birch, willows) on wet soils. Retaining larger buffer widths in discharge zones could also be compensated by reducing widths in other areas. Taken together, variable widths of riparian buffers, with wider retained forests on wet riparian hotspots and narrower buffers on areas with lower sensitivity, and the implementation of partial cuts would benefit important ecosystem services (water quality, biodiversity) without necessarily incurring costs from a wood production standpoint.

Variable riparian buffer widths can be effectively designed using hydromapping (Kuglerová et al. 2014a). By tracking the flow of water within riparian forests, forest managers can not only retain forests in areas of high ecological and biogeochemical significance but also mitigate the negative effects of logging and soil perturbation on wet areas, such as changed groundwater pathways, increased siltation and export of metals including methyl mercury (Bishop et al. 2009; Kuglerová et al. 2014a).

## Tools for water policy of future forests

The boreal forest comprise a mosaic of different soil characteristics, interspersed by scattered wetlands and lakes, and transected by numerous streams and rivers. At the same time, boreal forests in many parts of the world also represent managed semi-natural landscapes, with large-scale industrialized forestry (Rist et al. 2014). These dominant characteristics of the boreal zone present several challenges for forest managers because forest production has to be optimized in ways which do not compromise water quality. However, new technologies provide opportunities to meet this challenge through development of tools that can be implemented into every-day planning. Based on high-resolution LiDAR data that are becoming increasingly available together with modern computerized harvesters, it is now possible to implement automated methods for optimizing wood extraction and transport from the forest. Here, we have introduced the idea of hydromapping, which offers a cost-effective operational technique to minimize physical impacts on soils in sensitive areas and reduce negative effects on water quality. The new management approaches we suggest will require more input from scientists related to how intrinsic properties of forest ecosystems (e.g., landscape position, slope, underlying geology, soil texture etc.) influence both forest growth and the degree of sensitivity to various harvesting practices.

Maintaining or even increasing biomass production in boreal forests does not necessarily mean that other ecosystem services such as biodiversity will be unduly impacted, or that we will jeopardize either long-term soil sustainability or cause unacceptable deterioration of water quality. The state-of-the-art knowledge of water flow pathways in the landscape can lead the way to new tools that are based on mechanistic understanding of ecosystem functioning, and landscape heterogeneity. However, the legal protection and policy regarding forest management in connection with protecting small headwater streams will also partly depend on how small and temporary waters are defined by authorities (Acuna et al. 2014). Because of the large total length of small headwater streams in relation to watershed area (Bishop et al. 2008), an increased protection of such streams could affect large areas of relatively productive forest land, which would decrease the overall biomass yield. So, while some argue that intermittent/headwater streams are essential to the integrity of entire stream networks, others argue that full protection of these systems will be too costly. We argue that one way to move this debate forward is to develop and apply these new hydromapping tools as a means to improve forest landscape sustainability and identify the landscape areas that are most important for water quality protection.
